# Production of cadmium sulfide quantum dots by the lithobiontic Antarctic strain *Pedobacter* sp. UYP1 and their application as photosensitizer in solar cells

**DOI:** 10.1186/s12934-021-01531-4

**Published:** 2021-02-10

**Authors:** V. Carrasco, V. Amarelle, S. Lagos-Moraga, C. P. Quezada, R. Espinoza-González, R. Faccio, E. Fabiano, J. M. Pérez-Donoso

**Affiliations:** 1 BioNanotechnology and Microbiology Laboratory, Center for Bioinformatics and Integrative Biology, Facultad de Ciencias Biológicas, Universidad Andres Bello, Av. República 239, 8370146 Santiago, PC Chile; 2 Biochemistry and Microbial Genomics Department, Instituto de Investigaciones Biológicas Clemente Estable, Av. Italia 3318, 11600 Montevideo, PC Uruguay; 3 Centro Integrativo de Biología y Química Aplicada (CIBQA), Universidad Bernardo O’Higgins, General Gana 1702, 8370993 Santiago, PC Chile; 4 Departamento de Ingeniería Química, Biotecnología y Materiales, Facultad de Ciencias Físicas y Matemáticas, Universidad de Chile, Santiago, Chile; 5 Departamento de Experimentación y Teoría de la Estructura de la Materia y sus Aplicaciones, Facultad de Química, Universidad de la República, Av. Gral. Flores 2124, 11800 Montevideo, PC Uruguay

**Keywords:** Antarctica, Endoliths, Lithobionts, Nanoparticle biosynthesis, Cadmium sulfide nanoparticles, QDSSC, *Pedobacter*

## Abstract

**Background:**

Microbes are present in almost every environment on Earth, even in those with extreme environmental conditions such as Antarctica, where rocks may represent the main refuge for life. Lithobiontic communities are composed of microorganisms capable of colonizing rocks and, as it is a not so well studied bacterial community, they may represent a very interesting source of diversity and functional traits with potential for biotechnological applications. In this work we analyzed the ability of Antarctic lithobiontic bacterium to synthesize cadmium sulfide quantum dots (CdS QDs) and their potential application in solar cells.

**Results:**

A basaltic andesite rock sample was collected from Fildes Peninsula, King George Island, Antarctica, and processed in order to isolate lithobiontic bacterial strains. Out of the 11 selected isolates, strain UYP1, identified as *Pedobacter*, was chosen for further characterization and analysis due to its high cadmium tolerance. A protocol for the biosynthesis of CdS QDs was developed and optimized for this strain. After 20 and 80 min of synthesis, yellow-green and orange-red fluorescent emissions were observed under UV light, respectively. QDs were characterized through spectroscopic techniques, dynamic light scattering analysis, high-resolution transmission electron microscopy and energy dispersive x-ray spectroscopy. Nanostructures of 3.07 nm, composed of 51.1% cadmium and 48.9% sulfide were obtained and further used as photosensitizer material in solar cells. These solar cells were able to conduct electrons and displayed an open circuit voltage of 162 mV, a short circuit current density of 0.0110 mA cm^−2^, and had an efficiency of conversion up to 0.0016%, which is comparable with data previously reported for solar cells sensitized with biologically produced quantum dots.

**Conclusions:**

We report a cheap, rapid and eco-friendly protocol for the production of CdS QDs by an Antarctic lithobiontic bacterium, *Pedobacter*, a genus that was not previously reported as a quantum dot producer. The application of the biosynthesized QDs as sensitizer material in solar cells was validated.

## Background

The unique physicochemical properties that nanoparticles (NPs) exhibit make them a fascinating material to be used in the development of diverse technological products, giving rise during the last years to a huge expansion in nanomaterial related studies [1, 2]. In that sense, metal based NPs have been used in electronic/optic devices, biomedicine (diagnostic and therapeutic), energy, and control of microorganisms, among other uses [1, 3–6]. Particularly, QDs are a type of metal based NPs of about 1 to 6 nm [7], generally composed by elements of groups II–IV or II–V of the periodic table [8]. Size and composition of QDs, through a quantum confinement effect [9], give them distinctive optoelectronic properties such as a broad absorption and narrow and tunable emission spectra with strong luminescence [9, 10]. QDs have been used in diverse technological applications such as solar cells [11] and fluorescent biological labels with medical interests [12].

Quantum dots sensitized solar cells (QDSSCs) are special devices capable of harvesting sunlight for energy conversion by employing fluorescent semiconductor QDs [13]. Sunlight constitutes the most abundant renewable source of energy, and during the last decades photovoltaic technology has emerged as a promising source of “green” energy [14]. Particularly, high performance and relatively simple fabrication of QDs makes them good candidates as harvesting-materials in solar cells. Efficiencies of QDSSCs (0.003–10%) [1, 15] are still not as high as silicon solar cells (around 25%) or as solar cells constructed with other semi-conductor materials [16], therefore investigation aiming at improving QDSSCs’ materials, fabrication and efficiency is an active field of research. In this context, the development of photovoltaic technologies based on biologically produced materials is an emerging field with tremendous potential.

QDs are generally produced through top-down or bottom-up chemical and physical nano-manufacturing protocols [17]. Chemical synthesis requires specific facilities with complex operational conditions such as high temperatures, high pressures, and inert environments. Moreover, expensive high purity starting materials, toxic organic solvents and acidic or basic chemicals are required [18, 19]. In order to mitigate these disadvantages, biomimetic synthetic strategies are being developed as these generally use milder synthetic conditions and employ biological molecules which not only make synthesis simpler, cheaper, and eco-friendly, but also give QDs an increased stability and biocompatibility suitable for biological applications [20–22].

In the last few years, there has been an increased interest in biological synthesis of QDs and the use of microorganisms as biofactories. Biological synthesis requires mild conditions of pressure and temperature, and provides the possibility to scale-up the production and to tune the characteristics of the NPs by changing cell culture conditions or by genetic engineering [17, 23]. Little is known about the process of CdS QDs biosynthesis, and to date, the biological molecules involved, the molecular mechanisms, and the biological role of the process are not completely known.

Maintaining appropriate metal homeostasis is crucial for microorganisms and several systems are involved in controlling intracellular metal concentration [24–26]. Biotransformation of heavy metals into NP has been suggested as one of the mechanisms exerted by microorganisms to deal with metals [27]. Intracellular or surface entrapped metals can be transformed to NPs by the action of different enzymes which usually involves reductases and the attachment of stabilizing peptides containing cysteine, methionine, arginine and lysine [28–30]. However, biosynthetic mechanisms are yet to be fully elucidated.

Biosynthesized CdS QDs are among the most well studied and characterized metal sulfide NPs due to their potential technological applications [31, 32]. It has been shown that some bacteria produce CdS NPs in the presence of Cd^2+^ and sulfide anions [8, 33, 34]. Usually these CdS NPs are coated with biomolecules providing final CdS NPs with increased stability, water solubility and/or distinctive biocompatibility characteristics [31, 35, 36]. In particular, the use of extremophiles for QDs production has been proposed as an alternative to generate semiconductor nanocrystals with novel properties. By using polyextremophile-halophilic and acidophilic bacteria, stable CdS QDs have been synthesized at high salt concentration and low pH, respectively [32, 37].

In this context, the low temperature biosynthesis of Cd-based QDs (CdS, CdTe and CdSe) by psychrotolerant Antarctic *Pseudomonas* strains has been recently reported [38, 39]. In inhospitable environments, like in Antarctica, rocks can be the main refuge for life [40–42]. Rocks are ubiquitous and harbor a very interesting and not much explored microbial community, the lithobionts. These organisms colonize either the outside (exposed surface and underside) or the inside (through cracks, pores and fissures) of the lithic substrate, and may alter it by the deposition of metals or by bio-weathering [42, 43]. The close interaction between microorganisms and the mineral substrate could contribute to biomineralization processes, thus favoring the existence of lithobiontic microorganisms with the capacity to produce metal nanostructures.

Taking these considerations in mind, in this work we analyzed the ability of an Antarctic lithobiontic bacterium to biosynthesize CdS QD and assessed their potential application in QDSSCs.

## Results

### Selection, characterization and identification of *Pedobacter* sp. UYP1

With the aim to select the most promising strain for CdS QD synthesis from among the collection of Antarctic bacterial lithobionts, we evaluated their ability to tolerate the presence of CdCl_2_ and to produce H_2_S. Out of 11 bacterial lithobionts analyzed, strain UYP1 was selected for further work as it was the only one capable to tolerate 100 µM CdCl_2_ and was able to produce H_2_S (Additional file 1: Dataset S1). According to the determination of Minimal Inhibitory Concentration (MIC), strain UYP1 was able to grow up to 5 mM CdCl_2_, while higher concentrations were detrimental (Additional file 2: Dataset S2). UYP1 was also able to grow in the presence of high concentrations of manganese, iron, zinc, copper and nickel (Additional file 3: Dataset S3) indicating that UYP1 is tolerant to multiple metals.

According to 16S rRNA gene analysis, the UYP1 isolate belongs to the genus *Pedobacter*. The sequence analysis of the UYP1 isolate revealed a pairwise similarity higher than 95% with 15 *Pedobacter* type strains (Additional file 4: Dataset S4). Among them, a 99.7% pairwise similarity with only 4 mismatches in 1382 bp was determined with *Pedobacter crioconitis*. Besides, the analysis revealed a 98.19 and 98.12% pairwise similarity with *Pedobacter himalayensis* and *Pedobacter lusitanus*, respectively. It is interesting to note that most similar sequences belonged to psychrophilic bacteria, e.g.: A37 type strain and KOPRI 25599 were isolated from alpine cryoconites, BG5 and ANT H31B from Antarctica, glbl10 from glacier ice in Germany, and R20-57 from Austria alpine soils (Additional file 5: Dataset S5). Phylogenetic analysis of 16S rRNA also grouped UYP1 with other species of *P. cryoconitis*, but low bootstrap values obtained did not allow phylogenetic differentiation with *Pedobacter lusitanus*, *Pedobacter himalayensis*, *Pedobacter hartorius* or *Pedobacter westerhofensis*. Based on these results we named the strain as *Pedobacter* sp. UYP1.

### Production of H_2_S by *Pedobacter* sp. UYP1

In general, most methods to biosynthesize metal sulfur (MeS) NPs described to date require the addition of sulfur-containing molecules with high affinity for Cd^2+^ such as antioxidant thiols or the volatile sulfur compound H_2_S. Some bacteria are capable of releasing hydrogen sulfide (H_2_S) as a strategy to trap exogenous cadmium to form less toxic insoluble metal-sulfides, and this ability has been widely used in the biosynthesis of QDs during the last few years [8, 33, 44, 45]. Cysteine can be a sulfur source for H_2_S production [46], therefore we evaluated the ability of UYP1 to produce H_2_S when supplemented with cysteine. In these conditions UYP1 was capable of producing H_2_S, evidenced as a black precipitate in the lead acetate method (Additional file 6: Dataset S6).

### CdS QDs biosynthesis by *Pedobacter* sp. UYP1

**Fig. 1:**
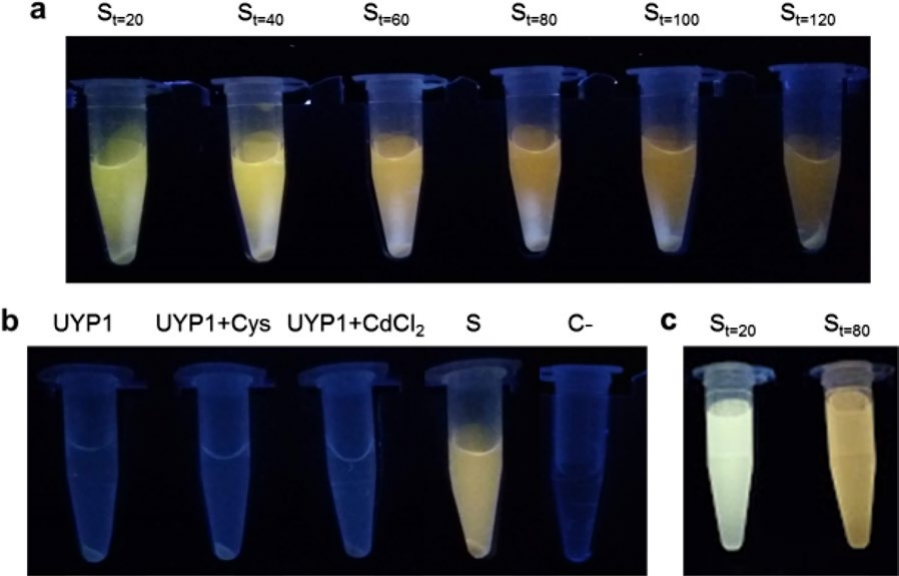
CdS QDs biosynthesis by *Pedobacter* sp. UYP1. **a** Biosynthesis of fluorescent nanoparticles through time. Cell suspensions were pelleted, and reaction tubes were exposed to UV in order to follow QD formation by the fluorescence presented. **b** Control conditions and biosynthesis condition tubes. Note that the negative control tube has the biosynthesis conditions, but no bacterial cells were added. **c** Purified and concentrated CdS QDs

In order to assess whether UYP1 was able to synthetize CdS QDs from CdCl_2_ and cysteine, preliminary biosynthesis experiments were performed at 28 °C. When cultures were exposed to UV light, fluorescent emission was observed which is characteristic of QDs (data not shown) [36, 38, 39, 47]. Moreover, fluorescence was only observed in the supernatant and not in the cell pellet, which suggests extracellular synthesis of NPs. As shown in Fig. 1a, fluorescence emission changes from green to red when incubation time increases, a behavior that is typical to QDs and depends on nanocrystal size [22, 48–50]. No fluorescence was observed when only cells were present, neither when cells were supplemented with either cysteine or CdCl_2_, nor in the abiotic control (synthesis conditions without cells) (Fig. 1b). Yellow-green (t = 20 min) and orange-red (t = 80 min) supernatants (Fig. 1c) were collected for further steps of QDs purification, concentration and characterization.

### Characterization of CdS QDs biosynthesized by *Pedobacter* sp. UYP1

**Fig. 2:**
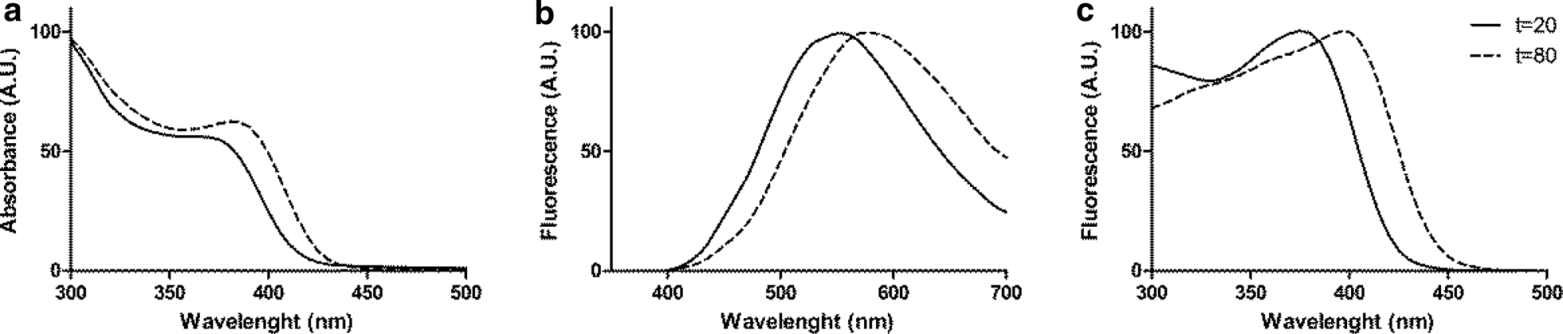
Spectroscopic characterization of CdS QDs synthesized by *Pedobacter* sp. UYP1. **a** Absorbance spectrum. **b** Fluorescence spectrum (λ_exc_ = 360 nm). **c** Excitation spectrum (λ_emi_ = 550 nm) of green-yellow QDs, biosynthesized after 20 min incubation (t = 20), and orange-red QDs (t = 80), biosynthesized after 80 min

**Fig. 3:**
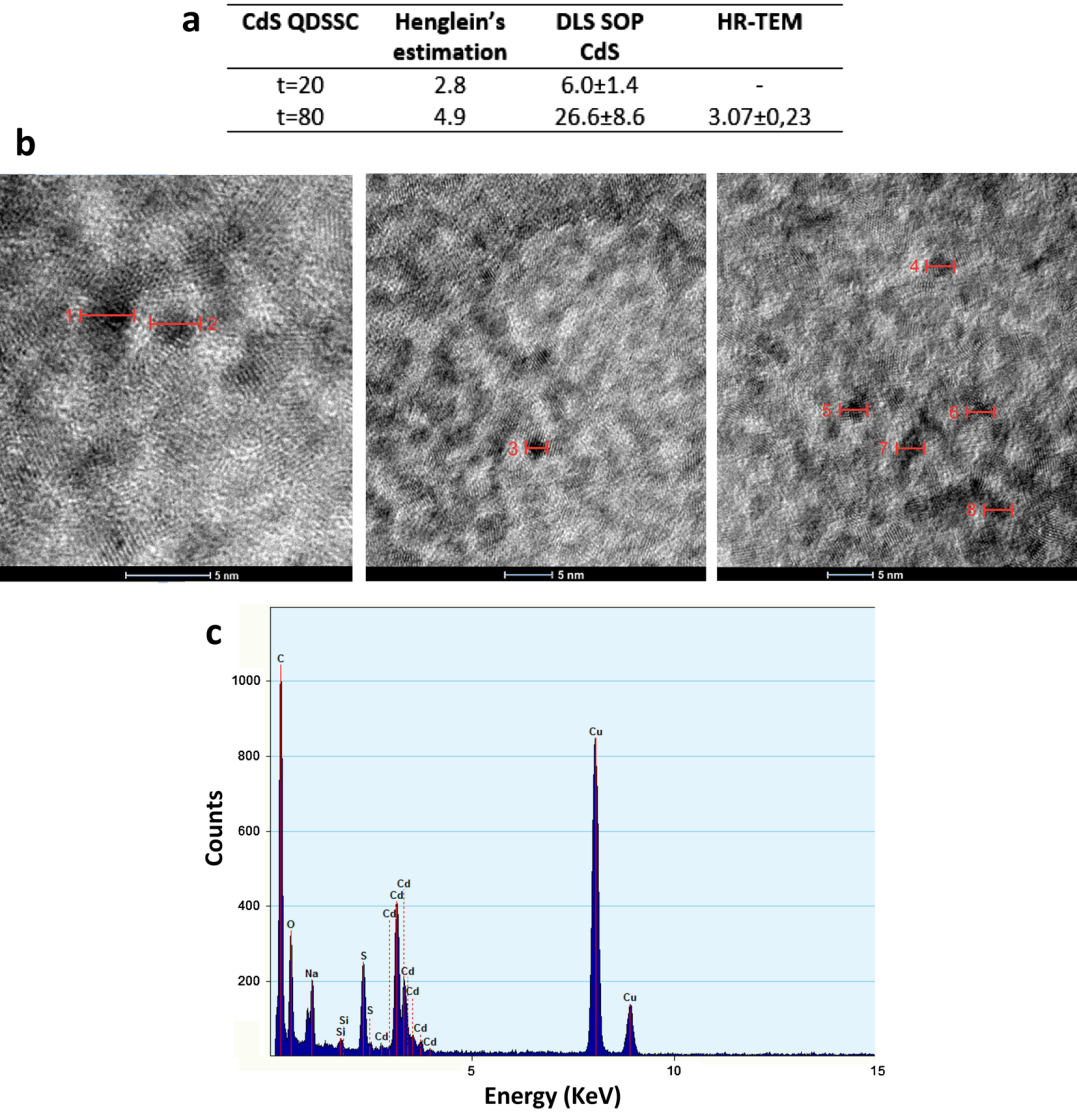
Size and chemical characterization of CdS QDs synthesized by *Pedobacter* sp. UYP1. **a** Size of biosynthesized QDs as determined by different methods. **b** Purified orange-red QDs visualized by HR-TEM. **c** EDX composition analysis of QDs

Purified QDs were analyzed by UV–visible spectroscopy. As shown in Fig. 2a, absorbance spectra were similar in shape for both, yellow-green and orange-red NPs, presenting peaks at 380 nm and 390 nm, respectively. As expected for CdS QDs, both spectra revealed high absorption levels in the UV range. Based on these spectra, and considering Henglein’s empirical model [51], diameter sizes of the QDs were estimated to be around 2.8 nm for NPs biosynthesized at 20 min, and 4.9 nm for NPs obtained after 80 min of incubation (Fig. 3a). After excitation at 360 nm, emission peaks were observed at around 550 nm and 585 nm for NPs biosynthesized at 20 and 80 min, respectively (Fig. 2b). This is in agreement with the colors observed in Fig. 1c. Excitation peaks at 380 nm and at 400 nm were observed for yellow-green and orange-red NPs, respectively (Fig. 2c).

To confirm the nanometric size of CdS NPs, a high-resolution transmission electron microscopy (HR-TEM) analysis was performed. Purified QDs produced by *Pedobacter* sp. UYP1 were 3.07 nm in size (Fig. 3b), which agrees with the 2.8 nm predicted by the Henglein’s equation. As derived from the DLS results (Fig. 3a), biosynthesized QDs contain a surface layer of biological molecules that increase their hydrodynamic diameter, a property that has been previously described for metal nanocrystals produced by bacteria [8].

Energy dispersive X-ray spectroscopy (EDX) analysis confirmed that biosynthesized CdS QDs were composed of 51.1% cadmium and 48.9% sulfide, while the amorphous matrix was mostly composed of organic matter with presence of carbon, oxygen, and other elements (Fig. 3c).

The Quantum Yield (QY) of biosynthesized NPs was determined to evaluate the efficiency of conversion of absorbed to emitted light occurring in the semiconductor nanostructure. A 12.23% QY was determined in QDs produced by *Pedobacter* sp. UYP1, a result that is similar to those reported in biological CdS NPs produced by other microorganisms [8, 37].

### CdS QDs biosynthesized by *Pedobacter* sp. UYP1 as photosensitizers in solar cells

Considering the excellent properties of the CdS QDs synthesized by UYP1 we decided to evaluate their potential application as photosensitizers in solar cells. For this purpose, QDSSCs were constructed and their performance was assessed. Solar cells sensitized with orange-red CdS QDs were able to conduct electrons and displayed an open circuit voltage (*V*_*OC*_) of 162 (mV), a short circuit current density (*I*_*SC*_) of 0.0110 (mA cm^−2^), and had 0.0016% of efficiency (*η*) (Table 1). In order to compare the performance of these biologically produced CdS QDs, results of previous studies that employed the same photosensitizer were included.

**Table 1:** QDSSC photovoltaic parameters

CdS QDs producing strain	Short current density Jsc (mA cm^−2^)	Open circuit voltage Voc (mV)	Efficiency η (%)	References
*Pedobacter* sp. UYP1	0.0110 ± 0.0005	162 ± 10	0.0016	This study
*Escherichia coli* BW25113	0.0819 ± 0.0035	279 ± 26	0.0080	[56]
*Escherichia coli* BW25113	0.0238 ± 0.0046	155	0.0027	[58]

CdS QDs synthetized by *Pedobacter* sp. UYP1 after 80 min were used as photosensitizers in solar cells. Four solar cells were constructed and the photovoltaic parameters obtained from the I–V curves. Average values and standard deviations for the assay are shown. Results from previous works were included for comparison

## Discussion

Lithobiontic communities are composed of microorganisms capable of colonizing rocks, which protect these organisms from extreme environmental conditions of wind, desiccation, high UV radiation and fluctuations in temperature; usual conditions in Antarctica. Based on the hypothesis that Antarctic rocks harbor a particular and not so explored bacterial community, in terms of diversity and physiology, the aim of this work was to identify and to characterize an Antarctic lithobiontic bacterium able to biosynthesize CdS QDs with promising properties as photosensitizer material for QDSSCs.

Out of 11 selected strains isolated from an Antarctic rock sample, *Pedobacter* sp. UYP1 was the most promising one for the biosynthesis of CdS QDs, as it exhibited high metal tolerance to several metallic salts, including CdCl_2_ (Additional file 2: Dataset S2 and Additional file 3: Dataset S3). Bacteria from the genus *Pedobacter* have been considered environmental superbugs, as many strains belonging to this genus harbor tolerance against diverse antimicrobial substances, hydrocarbons and heavy metals [52–54]. Moreover, UYP1 was a major producer of H_2_S compared with other Antarctic lithobionts assayed (Additional file 1: Dataset S1). The release of hydrogen sulfide by bacteria is one of the mechanisms involved in Cd tolerance due to the formation of insoluble metal-sulfides, including CdS NPs, which are less toxic to the cell. The alkaline pH of the borax-citrate buffer favors the deprotonation of H_2_S and the concomitant release of sulfide anions (S^2−^) able to trap exogenous Cd^2+^. Besides H_2_S, other sulfur-containing molecules such as glutathione, peptides and cysteine may be used as sulfur source for the production of CdS NPs [36, 45]. In the case of cysteine, the enzyme cysteine desulfhydrase has been found involved in sulfide production from cysteine [55]. In this work we found that cysteine favors the production of CdS NPs in *Pedobacter* sp. UYP1 (Fig. 1). Nevertheless, there are no available reports about the presence of cysteine desulfhydrase in *Pedobacter* spp. Future efforts should be done in order to confirm its presence in UYP1.

Our results show that under biosynthesis conditions and exciting at 360 nm, UYP1 produce extracellular CdS NPs after 20 min incubation with a maximum emission at yellow-green wavelengths, while after 80 min CdS NPs the maximum emission was in the orange-red wavelengths (Fig. 2b). It is well established that a main characteristic of QDs is a red-shift change in color fluorescence emission depending on time of incubation, which is a property directly correlated to an increase in the size of the nanocrystals [22, 48–50]. Size estimations by Henglein’s equation indicate that NPs biosynthesized after 20 min are smaller than those obtained after 80 min of incubation and also agree with the results obtained by DLS (Fig. 3).

Emission spectra obtained presented broad absorption peaks (Fig. 2b), indicative of a population of QDs with different sizes in the fractions analyzed. According to Gallardo et al. [38], peaks with a width bigger than 100 nm can be caused by the presence of organic compounds in the NPs. Results obtained by DLS also indicated a polydispersity in size of the NPs obtained. As DLS considers the hydrodynamic radius of the particles, sizes calculated with this method are usually bigger than those calculated with Henglein’s empirical model, which only considers the nanocrystal (metallic core). High polydispersity of NPs has been reported as a common trait of bacterially synthesized NPs [8, 36]. Moreover, it has been found that extracellular NPs are usually coated with organic compounds and are bigger than intracellular NPs [17]. The quantum yield determined in QDs produced by *Pedobacter* sp. UYP1 (12.23%) is in the range of the highest QYs determined in QDs produced by bacteria. QY values of 15.8, 23.53, and 21.04/7.81% have been previously determined in *E. coli*, *Halobacillus* sp., and an Antarctic *Pseudomonas* sp. [8, 37, 48].

In order to further characterize the CdS QDs, HR-TEM and EDX analysis were performed. Size of individual QDs’ metallic cores was in agreement with the one estimated through Henglein’s equation (Table 1). The results obtained from the chemical composition analysis confirmed that biosynthesized QDs were indeed composed of Cd and S in almost a 1:1 stoichiometric relationship. Furthermore, these NPs appeared to be covered with an organic layer of elements that have already been described in other biological synthesis of QDs [32], and that may probably derive from proteins produced by *Pedobacter* sp. UYP1. In this context, XRD characterizations of biosynthesized CdS NPs were performed, but no clear crystalline structures were determined (not shown). This is puzzling considering the fluorescent and structural properties determined for these QDs. Previous works in biosynthesized CdS QDs with similar properties to those described here (size, composition, QY, and emission), reported XRD results indicating the existence of crystalline structures [8, 36, 37, 56]. This result is most probably a consequence of the amorphous matrix of organic matter that covers the QDs produced by *Pedobacter* sp. UYP1.

Regarding the application of biosynthesized QDs as photosensitizer materials in the construction of solar cells, as far as we can tell, there had been only a few previous works. In Spangler et al. [57], an engineered strain of *Stenotrophomonas maltophilia* was used to produce PbS NPs, while in the works Órdenes-Aenishanslins et al. [56, 58], the bacteria employed was an *Escherichia coli* strain that was able to synthesize CdS QDs. To date, *Pedobacter* genus has not been used for this purpose. QDs biosynthesized by strain *Pedobacter* sp. UYP1 showed photovoltaic parameters that were in the same order of magnitude with those reported in the mentioned previous works. Photovoltaic parameters obtained using biological QDs as sensitizers are still too low for real applications. Different alternatives can be explored to improve their efficiency as sensitizers, such as passivation with another semiconductor, improving the characteristics of the device by thickening the TiO_2_ layer, or modifying the biosynthesized QDs. In this context, we recently developed two protocols to produce CdS/CdSe core shell QDs and ternary CdSAg_2_ QDs using *E. coli* cells. Both QDs were characterized, used to sensitize QDSSCs and the photovoltaic parameters determined. As expected, solar cells sensitized with core/shell and ternary QDs improved 10 times their efficiency when compared to CdS QDs (0.0222 v/s 0.00271%) [57, 59]. However, efficiencies obtained with these new methods are still far from those obtained with chemical QDs (1–3%).

Microorganisms inhabiting extreme environments have great potential in biotechnological applications, particularly those present in Antarctica that have been scarcely used to date. The present work validates the importance of isolating new microorganisms to be used in environmentally processes, such as the generation of energy. Photovoltaic parameters of solar cells using biological QDs reported to date are still not good enough to be used for energy production, however understanding their composition and how biological molecules define their properties will contribute to the development of sustainable green photovoltaic devices.

## Conclusions

This is the first report on QDs biosynthesis by a *Pedobacter* strain. *Pedobacter* sp. UYP1 constitutes a good candidate for biosynthesis because of its characteristics concerning high cadmium tolerance, H_2_S production, and growth in a wide range of temperatures. Here we describe a protocol for the production of CdS QDs from an Antarctic lithobiontic bacterium, which is rather cheap, rapid and eco-friendly. Biosynthesized NPs exhibited different colors due to their size and as a result of different reaction times. Their application in QDSSC was validated, although further optimization processes need to be done in order to obtain better photovoltaic parameters.

## Methods

### Isolation, selection and characterization of the strain used in this work

The strain used in this work was isolated from a basaltic andesite rock [59], collected in May 2014 during the 30th ANTARKOS Expedition, sponsored by the Uruguayan Antarctic Institute. The rock was located near General Artigas Station (62° 10′ 53.4″ S, 58° 54′ 32.1″ W) in the Fildes Peninsula of King George Island, South Shetland archipelago, Antarctica. The sample was aseptically collected and transported in a sterile plastic tube to the lab, being maintained at 4 °C during transport and until processing. A rock piece of 43 g was surface disinfected by immersion in 70% (v/v) ethanol for 1 min and then washed with sterile distilled water. Fifteen milliliters of sterile distilled water were added to the disinfected rock and the sample was vigorously vortexed during 10 min until visible disintegration. One hundred microliters of the suspension obtained was spread on R2A agar (Difco) containing 100 μg/mL of cycloheximide, to prevent fungal growth. Plates were incubated at 4 °C until bacterial colonies were clearly observed (about 30 days). From a total of 85 CFU (colony forming units) obtained, 11 colonies showing different morphologies were selected. Bacterial colonies were isolated by re-inoculating on R2A. Isolated colonies were grown at 4 °C in R2A broth (0.5 g yeast extract, 0.5 g proteose peptone, 0.5 g casamino acids, 0.5 g dextrose, 0.5 g soluble starch, 0.05 g MgSO_4_·7H_2_O, 0.3 g sodium pyruvate and 0.3 g K_2_HPO_4_, pH 7.2) and stored in 25% (v/v) glycerol at − 80 °C.

In order to select the most promising strains for CdS QD biosynthesis, Cd^2+^ tolerance of the 11 isolates was assessed in solid medium. Briefly, strains were grown in R2A broth at 4 °C until mid-exponential phase (OD_620nm_ of about 0.8–1) and 5 µL drops of the culture were plated in R2A agar supplemented with either 10 µM or 100 µM CdCl_2_. Plates were incubated at 4 °C and growth was evaluated during 30 days. For those isolates capable of growing with 100 µM CdCl_2_, tolerance was assessed in liquid medium. For this purpose, strains were grown at 4 °C in R2A broth until mid-exponential phase and 20-fold dilutions were made in R2A broth supplemented with either 10 µM or 100 µM CdCl_2_, cultures were grown at 4 °C with shaking and OD_620nm_ was measured periodically.

To determine minimal inhibitory concentration (MIC), the selected strain, named UYP1, was grown in R2A broth at 21 °C until mid-exponential phase and a 20-fold dilution was done in R2A broth supplemented with increasing concentrations of CdCl_2_. Cultures were grown at 21 °C for 72 h and growth was assessed as OD_620nm_. Maximal concentration of CdCl_2_ where the strain was still able to grow was considered as MIC.

It should be noted that strain UYP1 proved to be a psychrotolerant strain that grows within a wide range of temperatures. Even though it was isolated at 4 °C, its optimal growth temperature is around 21 °C. For practical purposes and due to the equipment available, we chose to continue performing our experiments at 21 and 28 °C.

### 16S rRNA gene sequencing and phylogenetic tree construction for strain UYP1

An almost complete sequence (1348 bp) of the 16S rRNA gene was obtained by colony PCR amplification using the universal primers 27F (5′-AGAGTTTGATCMTGGCTCAG-3′) and 1492R (5′-TACGGYTACCTTGTTACGACTT-3′) [60]. Amplicons were sequenced by Macrogen Inc. (South Korea). Forward and reverse sequences were assembled and curated using the DNA Baser V3 Sequence Assembler. Sequence obtained was deposited in the NCBI GenBank database with the Accession Number KU060818. Identification of bacterial genus was accomplished using the “Identify” tool at the EZBioCloud server 3.0 [61]. For the phylogenetic analysis, 16S rRNA gene sequences retrieved from the NCBI site corresponding to most similar type strains according to the EZ-Biocloud database and from most similar strains according to the RDP server (http://rdp.cme.msu.edu/) were used. Sequences were aligned using MUSCLE algorithm (http://www.drive5.com/muscle/) implemented in MEGA6 software (www.megasoftware.net). Evolutionary distances were calculated according to the Kimura two-parameter model with a gamma value of 0.45. Phylogenetic trees were reconstructed using the neighbor-joining algorithm. Robustness of the tree branches was estimated with 1000 bootstrap pseudoreplicates.

### H_2_S detection assay

Release of H_2_S by the bacterial strains was detected by visualization of lead sulfide (PbS) formation in presence of lead acetate as described by Shatalin et al. [46]. Briefly, strains were grown in R2A broth at 28 °C until mid-exponential phase in a 2 mL Eppendorf tube, 1 mM cysteine was added to the cultures and a filter paper moistened with a 0.1 M lead acetate solution and dried at 60 °C was attached under the cap of the tube and closed tightly. Release of H_2_S was determined after 72 h incubation at 28 °C as the formation of a black precipitate due to PbS formation. R2A broth (with and without cysteine) as well as bacterial cultures in medium without supplementation of cysteine were used as negative controls. The color intensity of the pixels was analyzed using ImageJ software.

### CdS QDs biosynthesis

UYP1 was grown in R2A broth at 28 °C until mid-exponential phase. Cells were harvested by centrifugation at 4100×*g* for 10 min, washed with distilled water and then concentrated 1.25× in borax-citrate buffer (30 mM borax, 15 mM citrate, pH 9.3). Aliquots were supplemented with 0.1 mM CdCl_2_ plus 1 mM cysteine, or with 0.2 mM CdCl_2_ plus 2 mM cysteine. Suspensions were incubated at 28 °C and QDs production was evaluated after 20, 40, 60, 80, 100 or 120 min. For that purpose, cells were pelleted by centrifugation at 27,670×*g* for 3 min and reaction tubes were exposed to UV light (λ_exc_ 360 nm) in a transilluminator. Cell suspensions without CdCl_2_ or without cysteine, or neither of these compounds were used as negative controls. A reaction control of borax-citrate buffer supplemented with CdCl_2_ and cysteine was also used as abiotic control.

### CdS QDs purification and concentration

For CdS QDs characterization, nanoparticles biosynthesized in an early and a late stage (20 min and 80 min, respectively) were selected. In order to purify and concentrate the extracellular NPs, intact cells were removed by filtration through a 0.22 μm pore size filter. QDs were then concentrated 10× by ultrafiltration using a 3.2 kDa cut-off filter (Amicon® Ultra 15 mL Centrifugal Filters).

### Characterization of biosynthesized nanoparticles

For spectroscopic characterization of purified NPs, their absorbance, emission and excitation spectra were determined at 25 °C by using a Synergy H1 (Biotek) multiwell plate reader. Absorbance spectra were recorded in the range of 300 nm to 500 nm. Emission spectra were obtained after excitation at 360 nm and recorded in the range of 300 nm to 700 nm, while excitation spectra were measured as the fluorescence emission at 550 nm and an excitation range from 300 to 500 nm.

The quantum yield (QY) of biosynthesized NPs was determined following the protocol previously described by Venegas, et al. [48] Briefly, CdS NPs were dissolved in distilled water, and also for fluorescein in ethanol (QY = 0.91) (Sigma-Aldrich, St. Louis, MO, USA). Different samples with absorbance values between 0.01 and 0.1 A.U. under excitation at 360 nm were prepared. Fluorescence spectra were recorded for obtaining the integrated fluorescence intensity (IFI). Then, IFI was plotted versus the absorbance of NPs’ solutions. In the comparative method, the QY is calculated using the slope of the line determined from the plot of the absorbance against the IFI. In this case, the QY can be calculated using: QYNPs = QYR[mNPs/mR][nNPs/n2R] − 1. Curves’ slopes (m) and refractive index of the solvent (n) (water: 1.333, and ethanol: 1.335) were used to calculate NPs QY by considering fluorescein as reference (R).

Dynamic Light Scattering (DLS) measurements were realized in 4 optical paths cuvettes using a Zetasizer Nano ZSP (Malvern Institute Ltd). In order to evaluate NPs size, high-resolution transmission electron microscopy (HR-TEM) was performed according to Ulloa et al. [32]. Briefly, a suspension of purified NPs was placed on a copper grid and analyzed with FEI Tecnai G2 F20 S-Twin microscope, operated at 200 kV. TEM images were processed and analyzed using Digital Micrograph 3.9.0 (Gatan Inc.) and The Gimp 2.4.0 software packages.

Chemical characterization of the NPs synthesized was performed by Energy-dispersive X-ray spectroscopy (EDX) coupled to HR-TEM (using a FEI Tecnai G2 F20 S-Twin microscope, operated at 200 kV). For these studies, 2 µL of the concentrated CdS QDs solution was added to a HC300-Cu grid and left to dry. TEM images were processed and analyzed with Digital Micrograph 3.9.0 (Gatan Inc.) and The Gimp 2.4.0 software packages.

### Fabrication of quantum dot sensitized solar cells (QDSSC)

Quantum dots sensitized solar cells were constructed as in the protocol described by Órdenes-Aenishanslins et al. [56] with minor modifications. Electrodes with an area of 20 × 20 × 2 mm size were used with fluorine doped tin oxide coated glass (FTO glass) TEC15, with surface resistivity of 13 Ω sq^−1^ and 82–84.5% of transmittance (Sigma-Aldrich). To remove organic contaminants, conductive glasses were sonicated in deionized water for approximately 10 min. The anode was prepared by deposition of a suspension of TiO_2_ nanoparticles over a FTO glass with two 35 μL coats through spin-coating (for a 1 cm^2^ active area) and by a subsequent sintering process at 465 °C for 25 min. The counter electrode (cathode) was build up by spin-coating 10 μL of a 50 μM H_2_PtCl_6_·6H_2_O solution in isopropanol over a FTO coated glass and by heating the glass at 400 °C for 20 min. The sensitization of the TiO_2_ film was done by direct absorption of the CdS QDs. Before the assembly of the cell, 14 μL of the electrolyte solution were added. The solution used was sulfide/polysulfide (S^2−^/S_*n*_^2−^) and it was prepared with 1 M Na_2_S, 0.1 M S and 0.1 M NaOH dissolved in ultrapure water. Measurements were performed using sun intensity irradiance (~ 100 mW cm^−2^ and AM1.5) as light source.

## Supplementary Information

**Additional file 1: Dataset S1.** Production of H_2_S and bacterial growth in medium with Cd^2+^ of the lithobiont collection.

**Additional file 2: Dataset S2.** Determination of CdCl_2_ MIC of strain UYP1. The bar plots represent the mean optical density (± standard deviation, SD) of the experimental conditions, each performed in triplicate.

**Additional file 3: Dataset S3.** Tolerance of strain UYP1 of different metal salts.

**Additional file 4: Dataset S4.** 16S rRNA similarity search results.

**Additional file 5: Dataset S5**. Phylogenetic analysis of strain UYP1. A Neighbor joining tree was constructed using 16S rRNA nucleotide sequences of fifteen *Pedobacter* type strains retrieved by EZBioCloud (highlighted in bold) and fifteen strains retrieved by RDP and downloaded from NCBI e-servers, as most similar in sequence with UYP1. *Pedobacter* sp. UYP1 phylogenetic position is indicated by an arrow. Accession numbers for all the strains are detailed in brackets.

**Additional file 6: Dataset S6.** H_2_S_(g)_ liberation assay. White filter paper was moistened with a 0.1 M lead acetate solution, air-dried and attached to the lid of tubes with different conditions. H_2_S_(g)_ liberation was therefore assessed according to the lead acetate method [46]. (a) Negative control (R2A medium) with or without 1 mM cysteine and *Pedobacter* sp. UYP1 strain grown in R2A medium, with or without 1 mM cysteine. (b) Relative intensity values obtained from the pixel intensity analysis of the images in a.

## Data Availability

The datasets supporting the conclusions of this article are included in the manuscript and Additional files.
