# Supplementary material for: Production of cadmium sulfide quantum dots by the lithobiontic Antarctic strain Pedobacter sp. UYP1 and their application as photosensitizer in solar cells
Source: Microb Cell Fact. 2021 Feb 10;20:41. doi: 10.1186/s12934-021-01531-4 (PMC7876818; doi:10.1186/s12934-021-01531-4)
Supplement: Supplementary file 1 — Additional file 1: Dataset S1. Production of H2S and bacterial growth in medium with Cd2+ of the lithobiont collection. [file 12934_2021_1531_MOESM1_ESM.docx]

| Strain | Cys (-)^a^ | Cys (+)^a^ | Growth in R2A +100 µM CdCl_2_^b^ |
| --- | --- | --- | --- |
|  |  | |  |
| UYP1 | 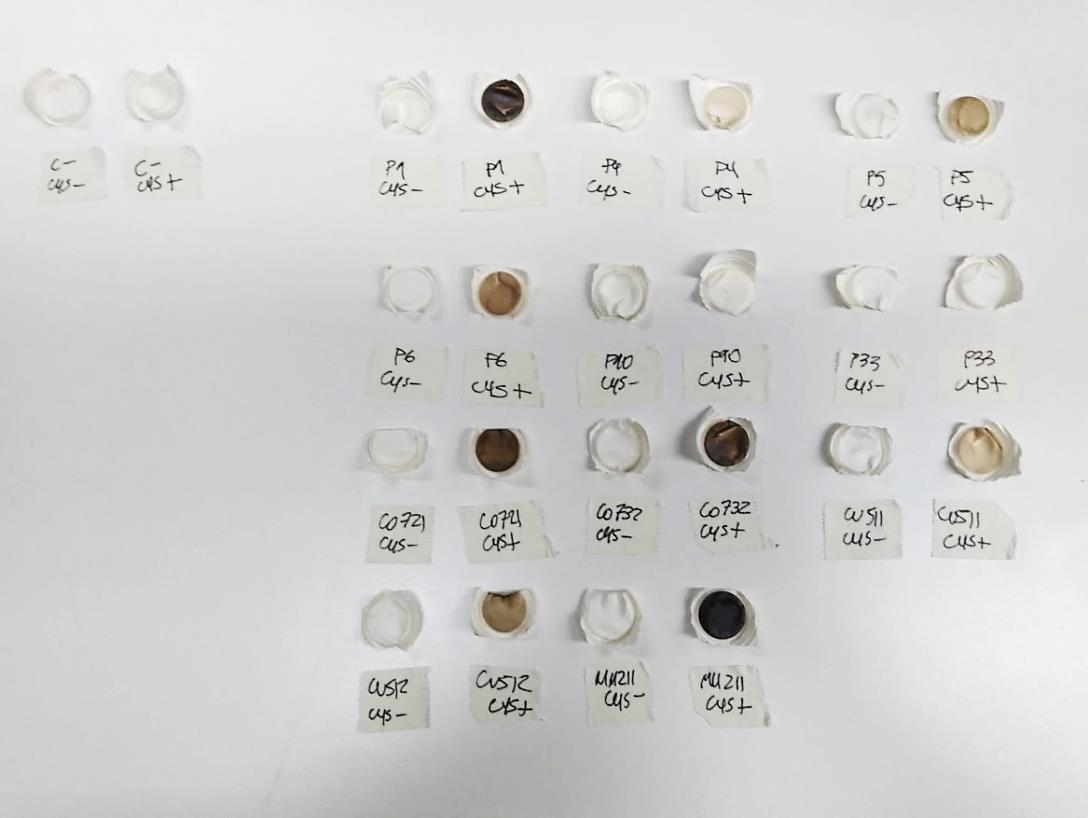 | | + |
| UYP4 | 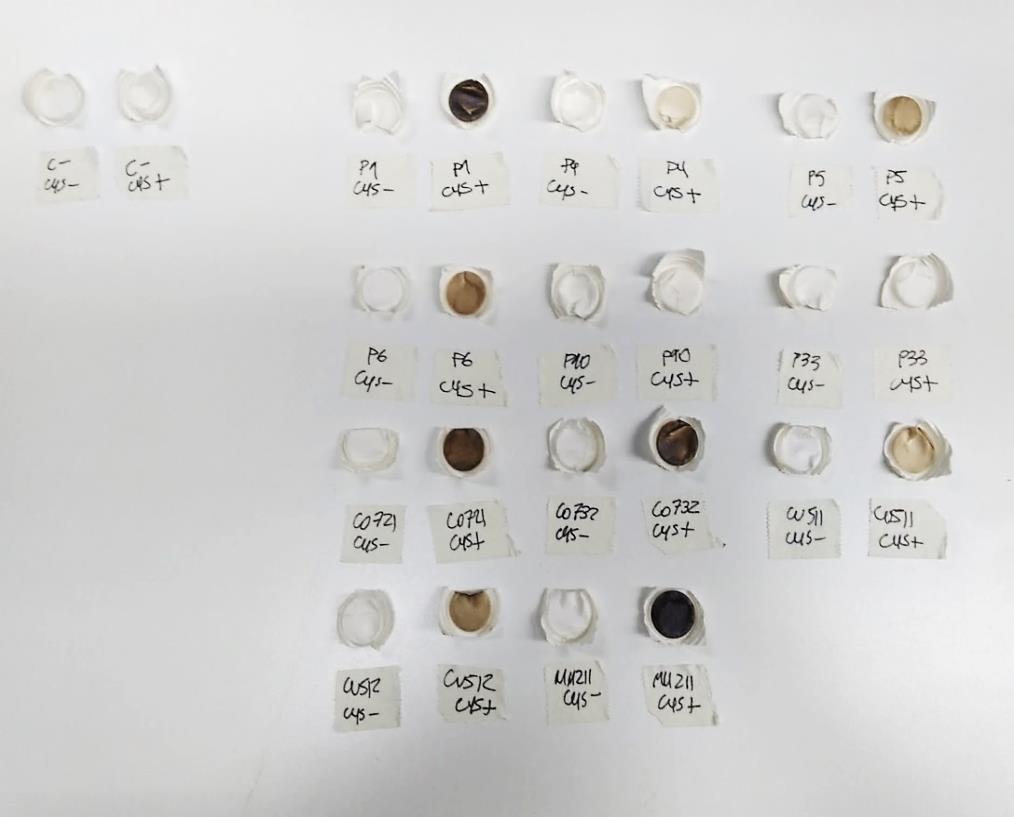 | | - |
| UYP5 | 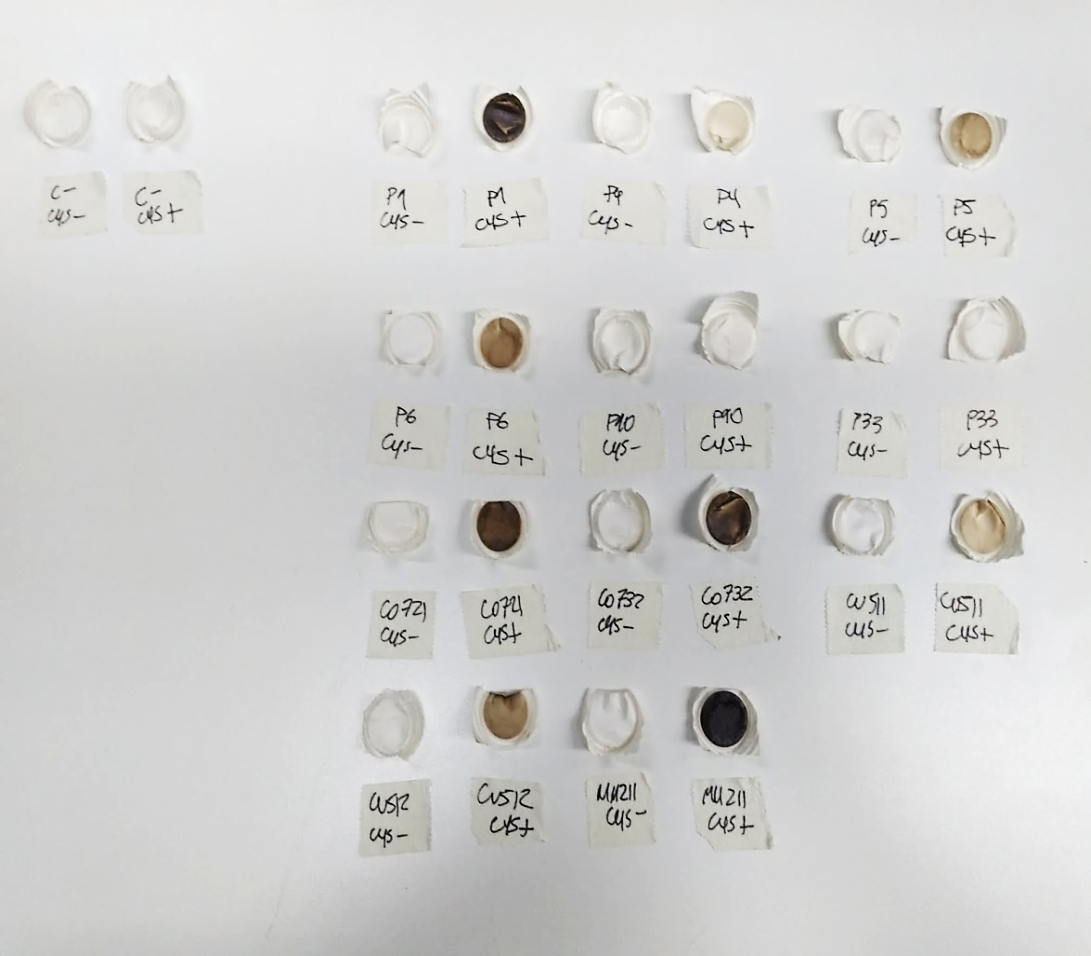 | | - |
| UYP6 | 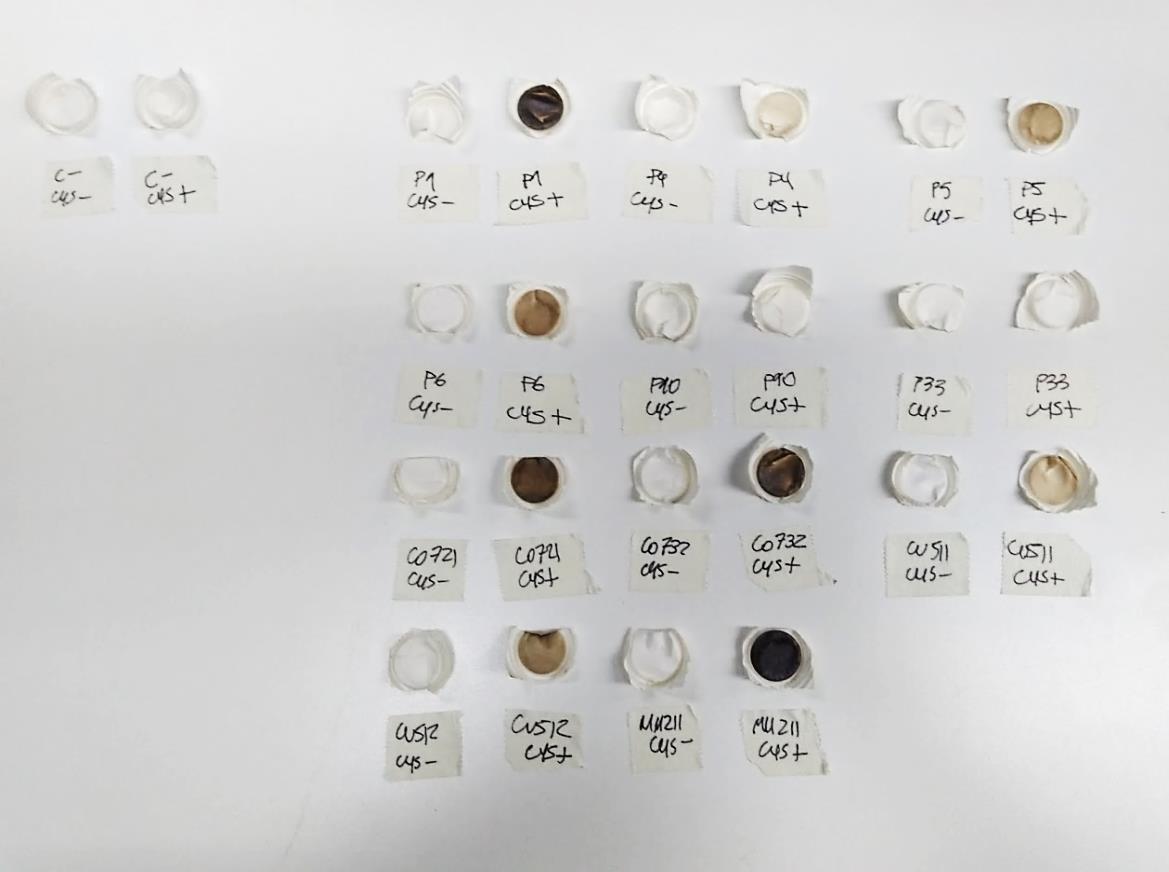 | | - |
| UYP10 | 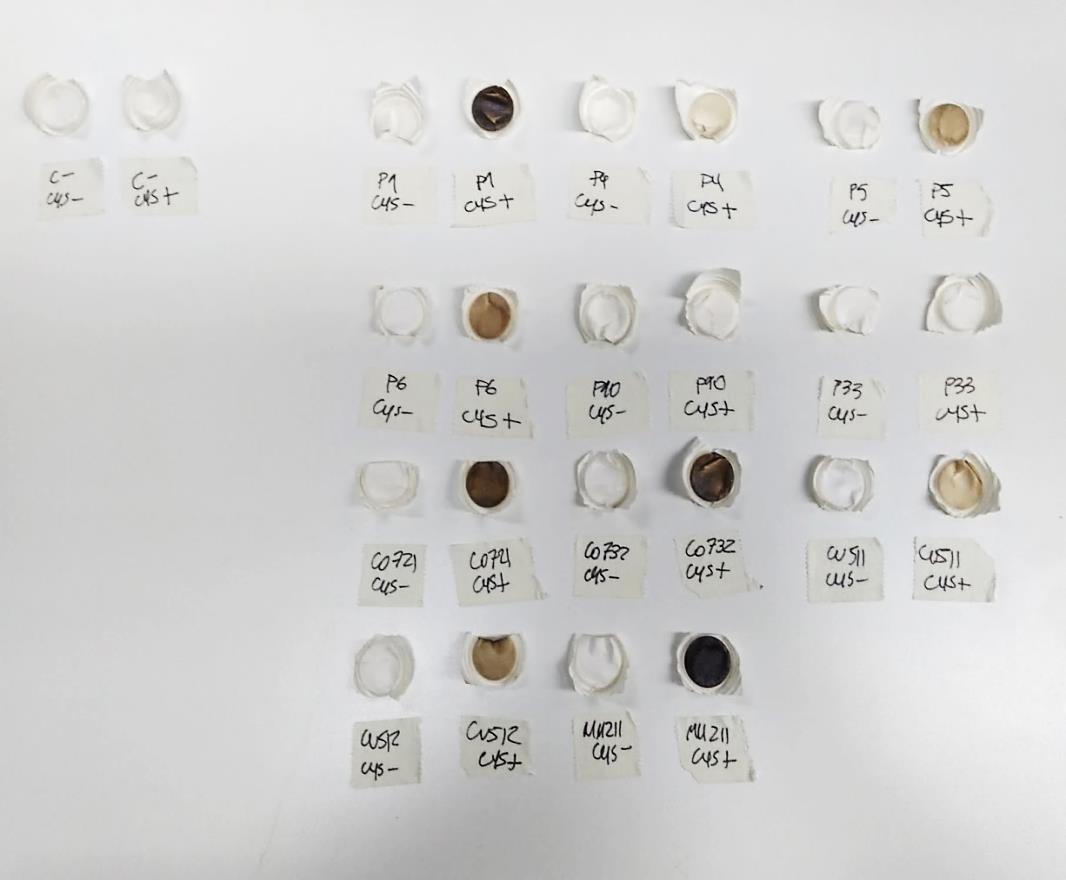 | | - |
| UYP33 | 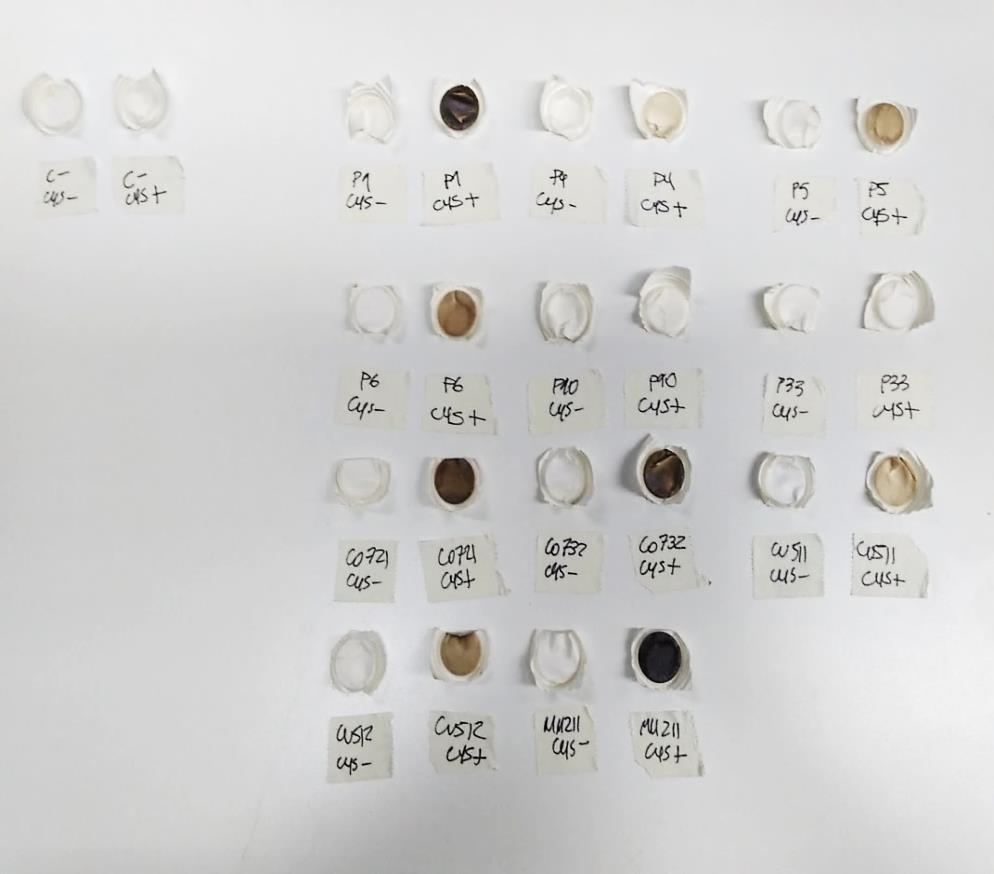 | | - |
| Co721 | 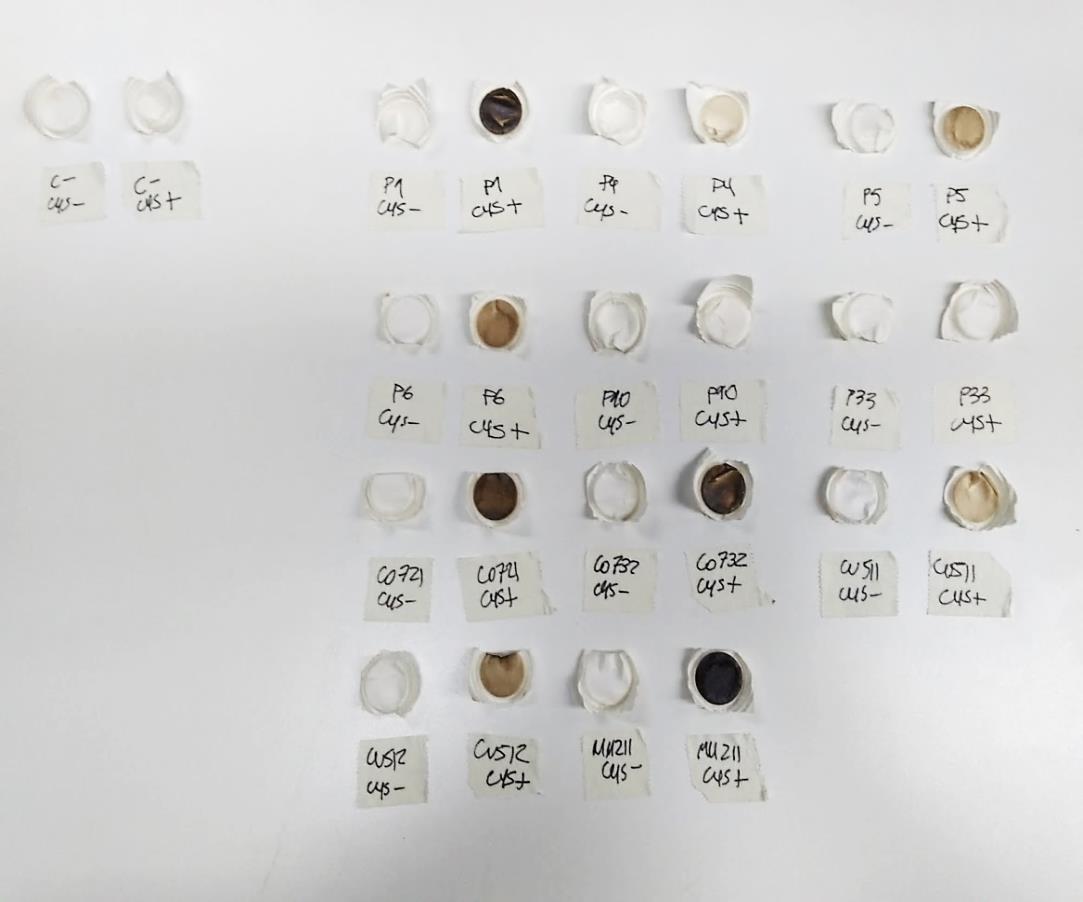 | | - |
| Co732 | 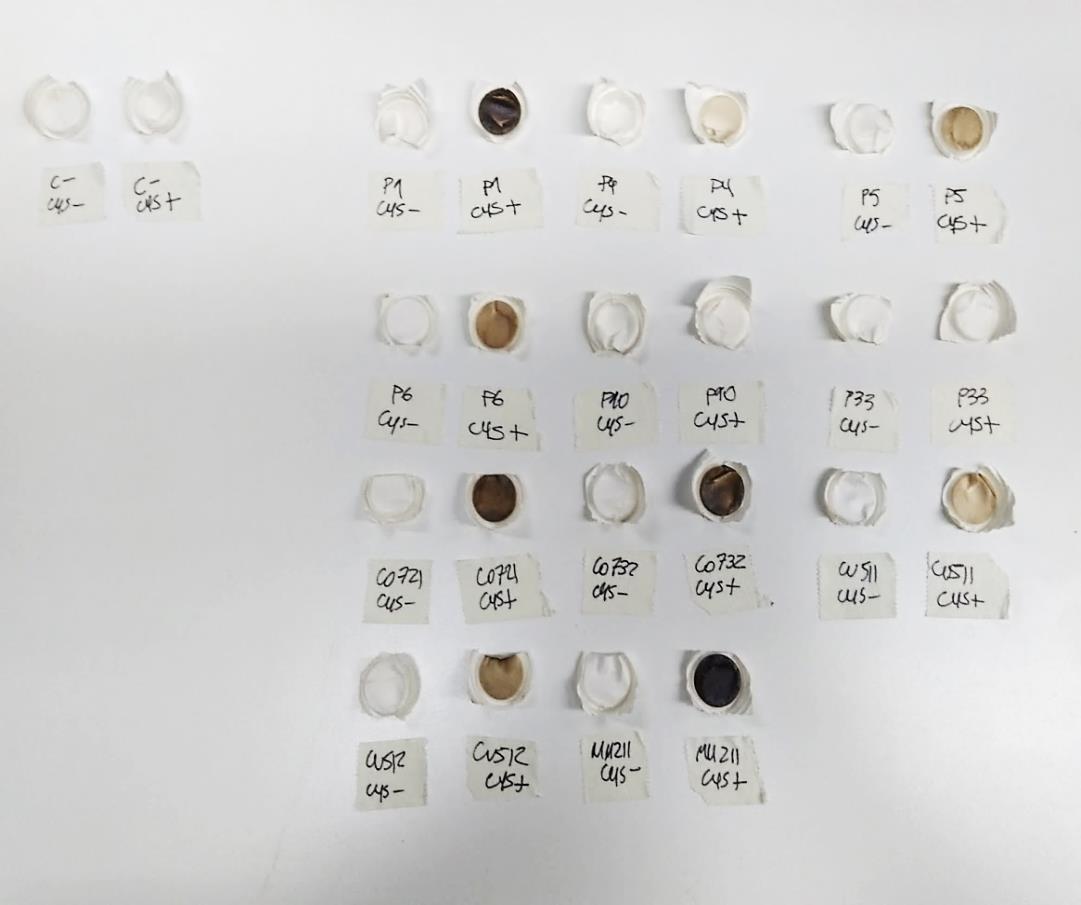 | | - |
| Cu511 | 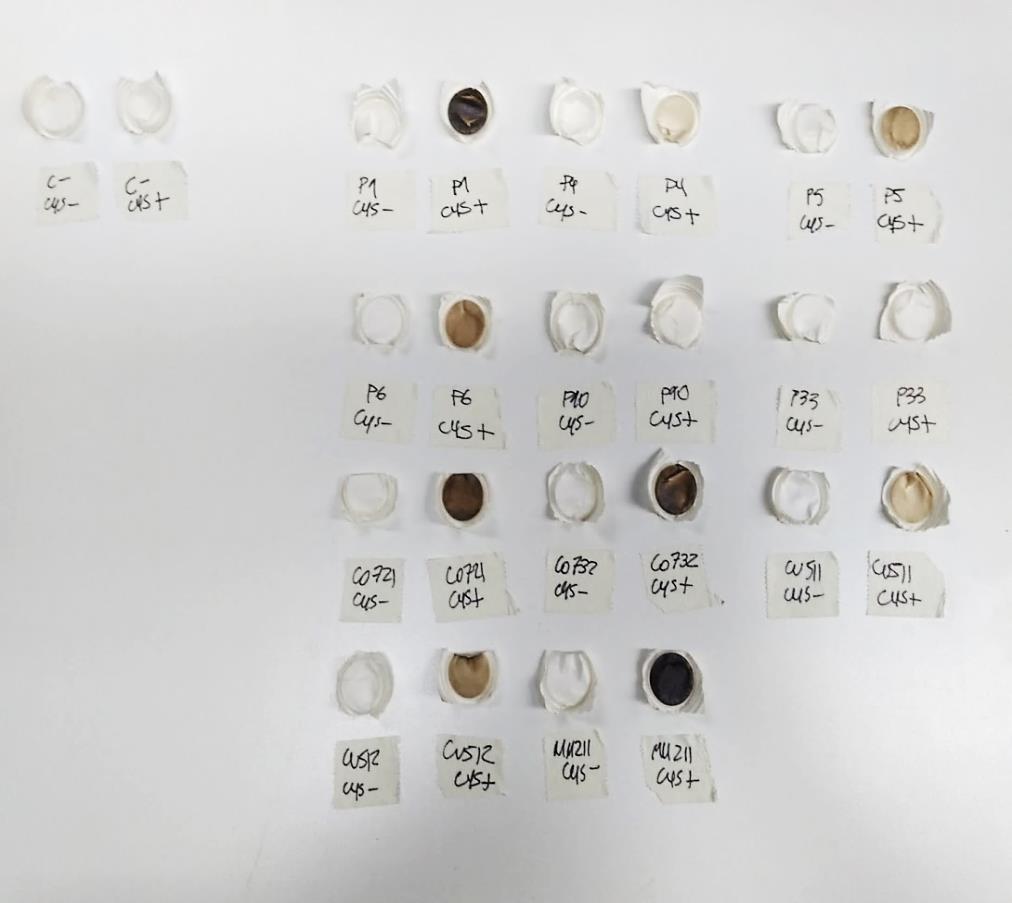 | | - |
| Cu512 | 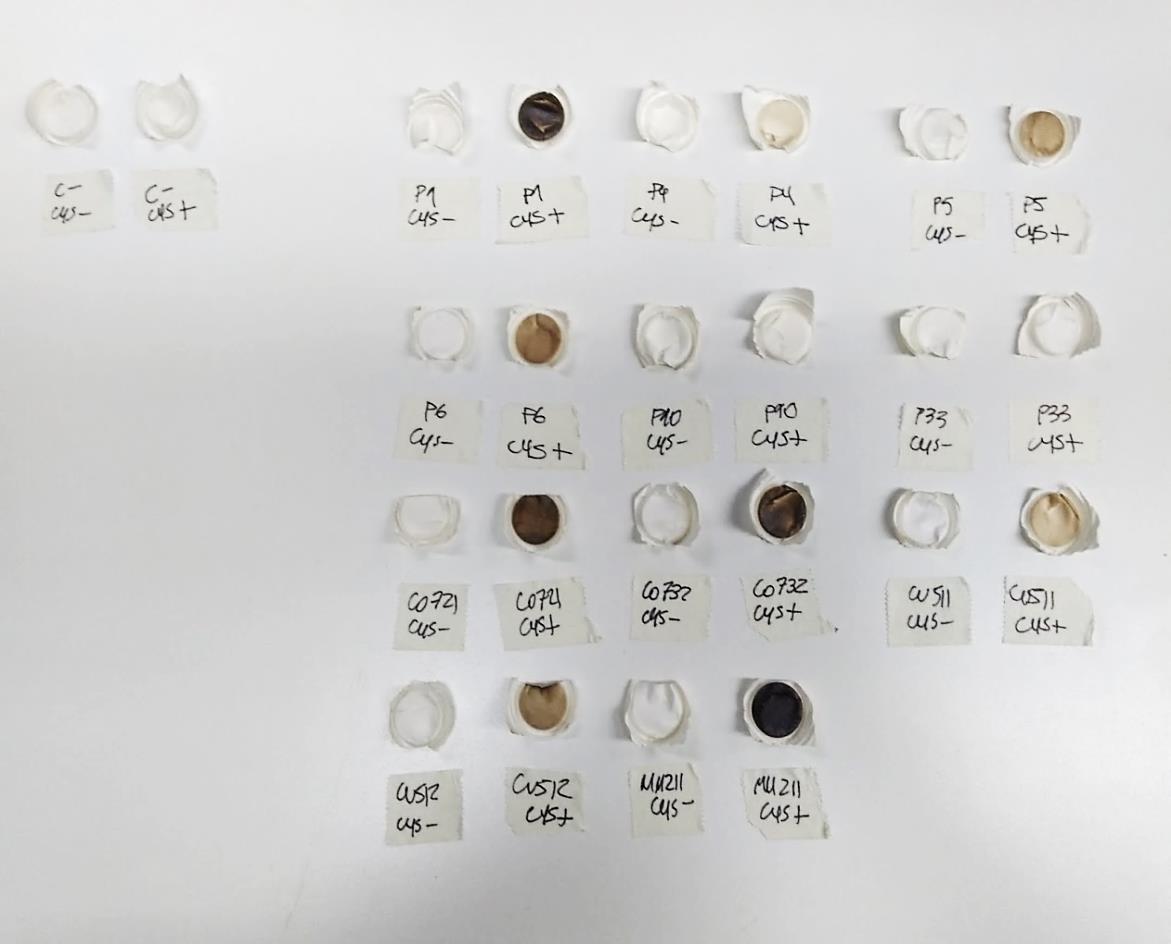 | | - |
| MM211 | 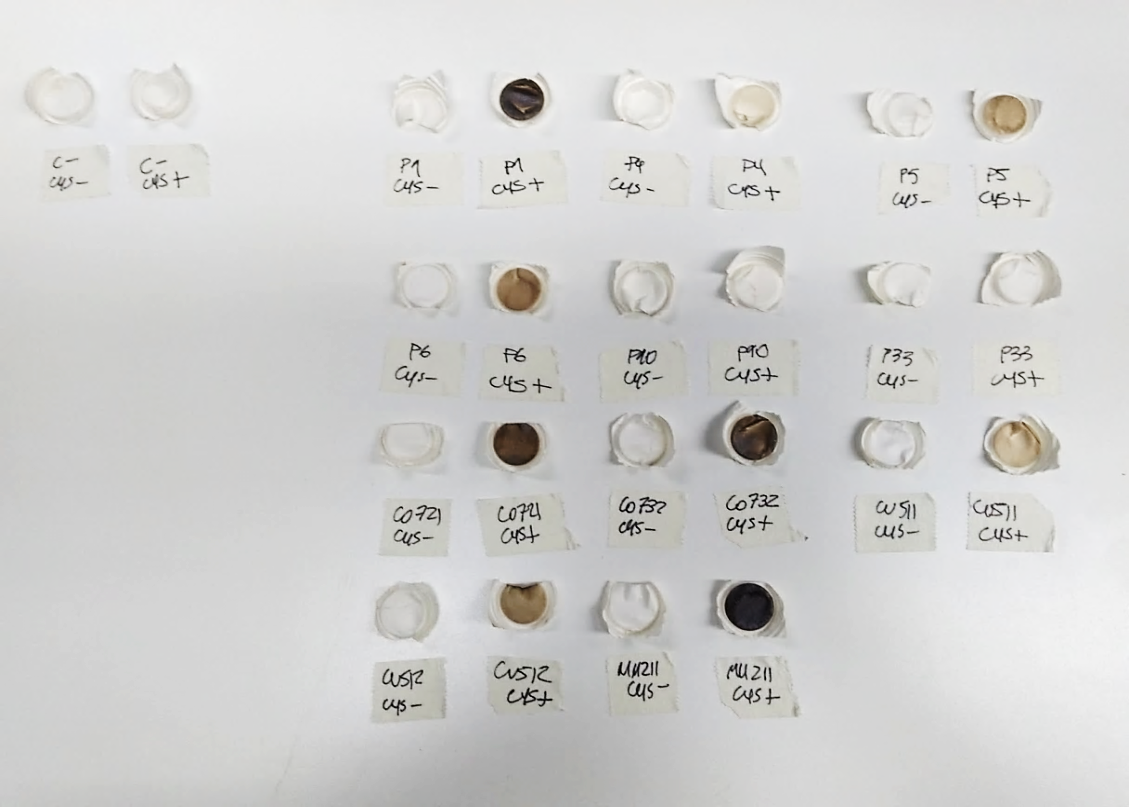 | | - |
| Control^c^ | 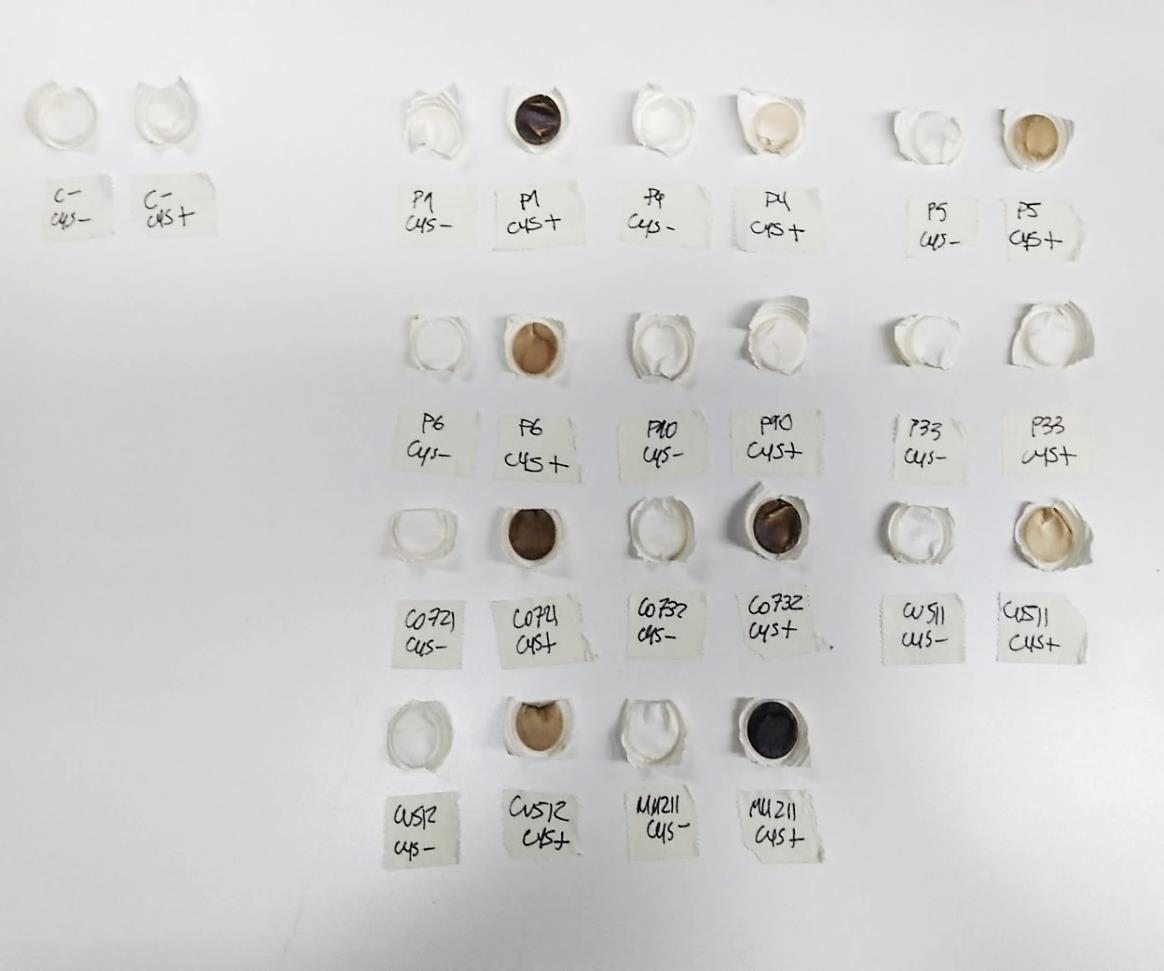 | | NA^d^ |

**Table S1.** Production of H_2_S and bacterial growth in medium with Cd^2+^ of the lithobiont collection

^a^ Cys (-), no cysteine was added to the medium; Cys (+), 1 mM cysteine was added to the medium

^b^ Bacterial growth in R2A solid medium with 100 µM CdCl_2_

^c^ Control condition without bacteria

^d^ Not applicable
