# Supplementary figures and images for: Production of cadmium sulfide quantum dots by the lithobiontic Antarctic strain Pedobacter sp. UYP1 and their application as photosensitizer in solar cells
Source: Microb Cell Fact. 2021 Feb 10;20:41. doi: 10.1186/s12934-021-01531-4 (PMC7876818; doi:10.1186/s12934-021-01531-4)

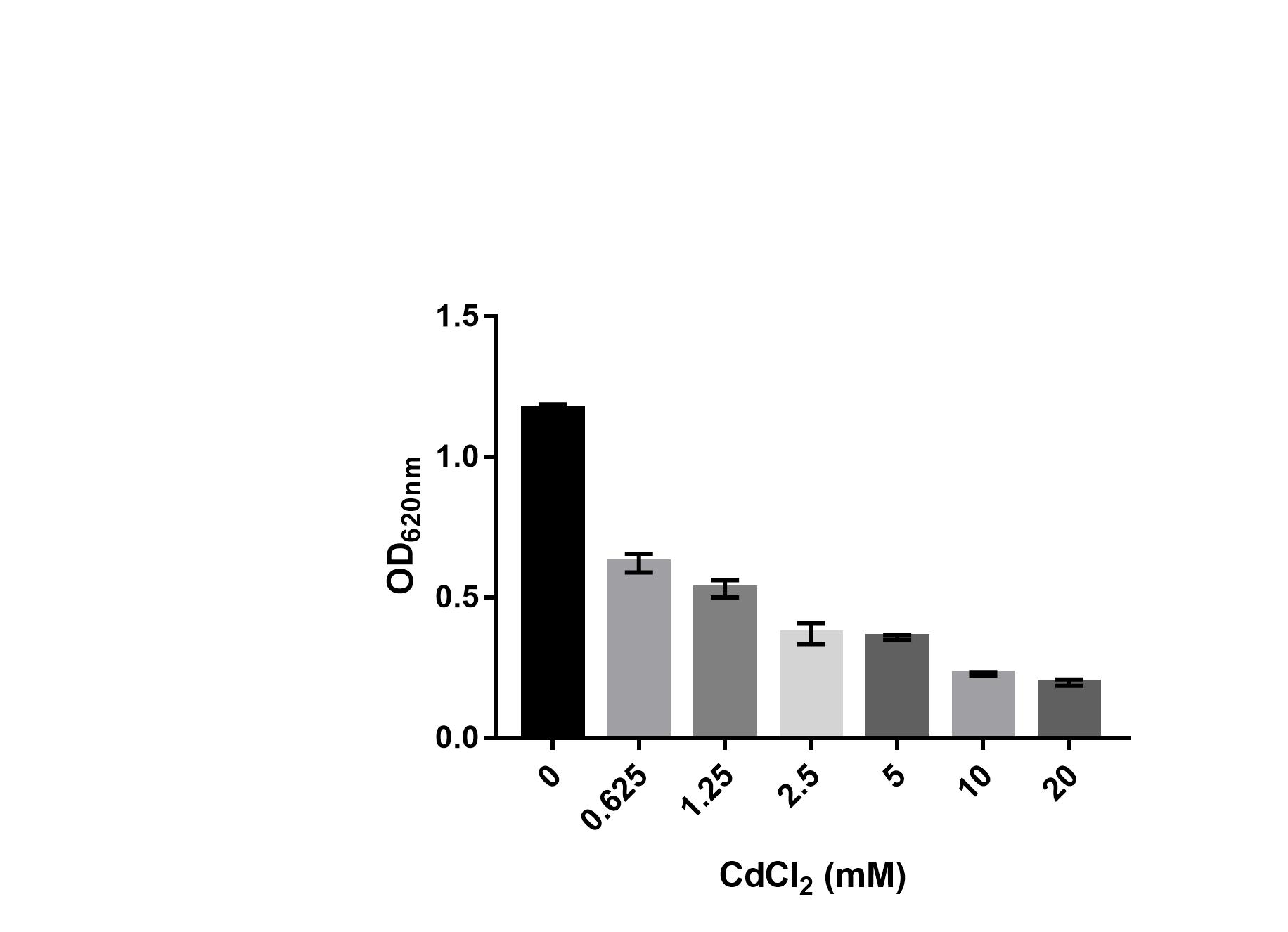

Supplement: Supplementary file 2 — Additional file 2: Dataset S2. Determination of CdCl2 MIC of strain UYP1. The bar plots represent the mean optical density (± standard deviation, SD) of the experimental conditions, each performed in triplicate. [file 12934_2021_1531_MOESM2_ESM.jpg]

## Slide 1
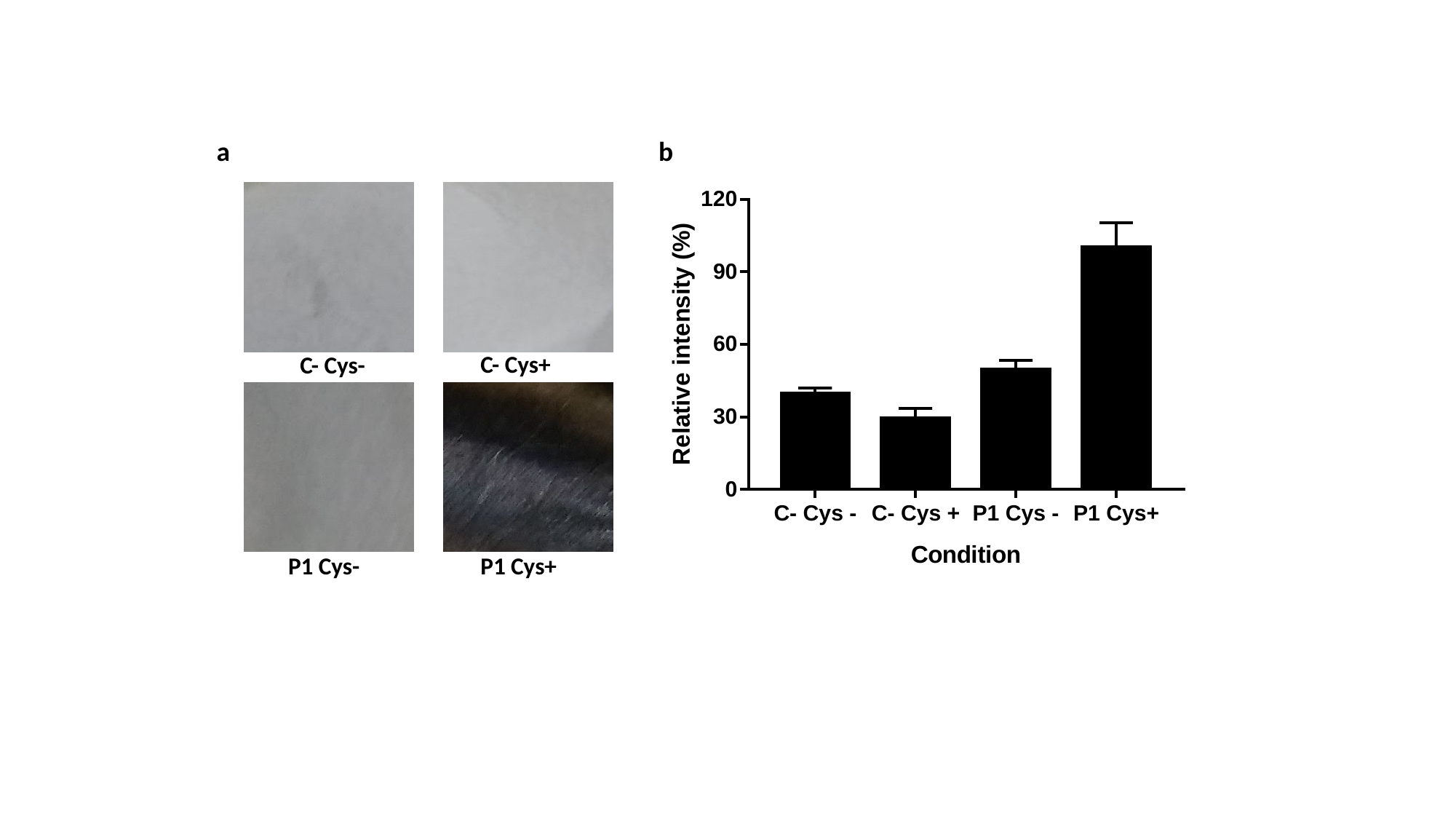

a
b
C- Cys+
C- Cys-
P1 Cys-
P1 Cys+

Supplement: Supplementary file 6 — Additional file 6: Dataset S6. H2S(g) liberation assay. White filter paper was moistened with a 0.1 M lead acetate solution, air-dried and attached to the lid of tubes with different conditions. H2S(g) liberation was therefore assessed according to the lead acetate method [46]. (a) Negative control (R2A medium) with or without 1 mM cysteine and Pedobacter sp. UYP1 strain grown in R2A medium, with or without 1 mM cysteine. (b) Relative intensity values obtained from the pixel intensity analysis of the images in a. [file 12934_2021_1531_MOESM6_ESM.pptx]
