# Supplementary material for: Production of cadmium sulfide quantum dots by the lithobiontic Antarctic strain Pedobacter sp. UYP1 and their application as photosensitizer in solar cells
Source: Microb Cell Fact. 2021 Feb 10;20:41. doi: 10.1186/s12934-021-01531-4 (PMC7876818; doi:10.1186/s12934-021-01531-4)
Supplement: Supplementary file 3 — Additional file 3: Dataset S3. Tolerance of strain UYP1 of different metal salts. [file 12934_2021_1531_MOESM3_ESM.docx]

**Dataset S3**

| **Metal Compound** | **MIC (mM)** |
| --- | --- |
| MnCl_2_ | >5 |
| ZnSO_4_ | >5 |
| CoCl_2_ | 0.5 |
| FeCl_3_ | 5 |
| CuSO_4_ | 1 |
| NiCl_2_ | 1 |
| CdCl_2_ | 5 |
