# Supplementary material for: Production of cadmium sulfide quantum dots by the lithobiontic Antarctic strain Pedobacter sp. UYP1 and their application as photosensitizer in solar cells
Source: Microb Cell Fact. 2021 Feb 10;20:41. doi: 10.1186/s12934-021-01531-4 (PMC7876818; doi:10.1186/s12934-021-01531-4)
Supplement: Supplementary file 4 — Additional file 4: Dataset S4. 16S rRNA similarity search results. [file 12934_2021_1531_MOESM4_ESM.pptx]

## Slide 1
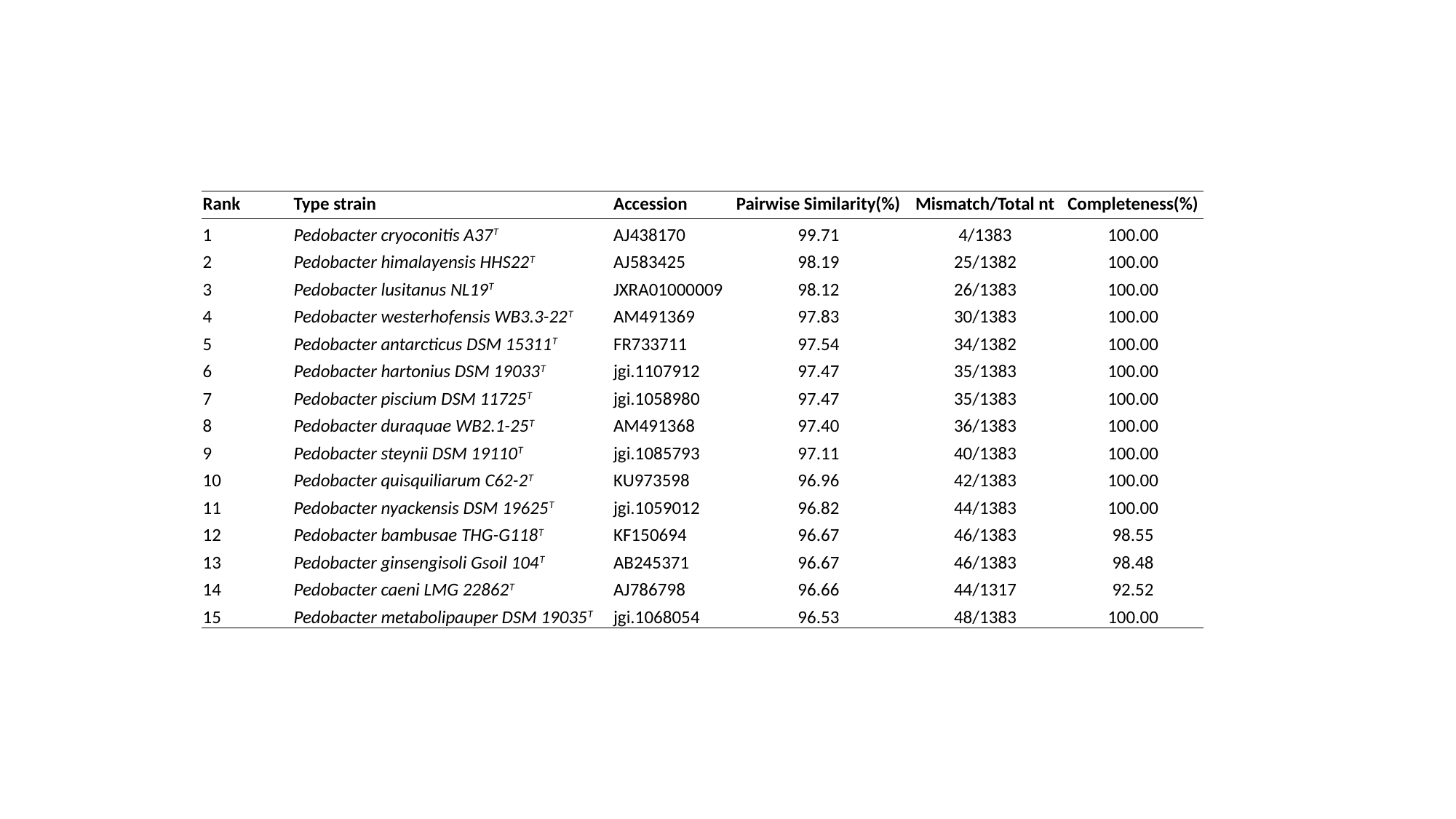

| Rank | Type strain | Accession | Pairwise Similarity(%) | Mismatch/Total nt | Completeness(%) |
| --- | --- | --- | --- | --- | --- |
| 1 | Pedobacter cryoconitis A37T | AJ438170 | 99.71 | 4/1383 | 100.00 |
| 2 | Pedobacter himalayensis HHS22T | AJ583425 | 98.19 | 25/1382 | 100.00 |
| 3 | Pedobacter lusitanus NL19T | JXRA01000009 | 98.12 | 26/1383 | 100.00 |
| 4 | Pedobacter westerhofensis WB3.3-22T | AM491369 | 97.83 | 30/1383 | 100.00 |
| 5 | Pedobacter antarcticus DSM 15311T | FR733711 | 97.54 | 34/1382 | 100.00 |
| 6 | Pedobacter hartonius DSM 19033T | jgi.1107912 | 97.47 | 35/1383 | 100.00 |
| 7 | Pedobacter piscium DSM 11725T | jgi.1058980 | 97.47 | 35/1383 | 100.00 |
| 8 | Pedobacter duraquae WB2.1-25T | AM491368 | 97.40 | 36/1383 | 100.00 |
| 9 | Pedobacter steynii DSM 19110T | jgi.1085793 | 97.11 | 40/1383 | 100.00 |
| 10 | Pedobacter quisquiliarum C62-2T | KU973598 | 96.96 | 42/1383 | 100.00 |
| 11 | Pedobacter nyackensis DSM 19625T | jgi.1059012 | 96.82 | 44/1383 | 100.00 |
| 12 | Pedobacter bambusae THG-G118T | KF150694 | 96.67 | 46/1383 | 98.55 |
| 13 | Pedobacter ginsengisoli Gsoil 104T | AB245371 | 96.67 | 46/1383 | 98.48 |
| 14 | Pedobacter caeni LMG 22862T | AJ786798 | 96.66 | 44/1317 | 92.52 |
| 15 | Pedobacter metabolipauper DSM 19035T | jgi.1068054 | 96.53 | 48/1383 | 100.00 |
