# Supplementary material for: Production of cadmium sulfide quantum dots by the lithobiontic Antarctic strain Pedobacter sp. UYP1 and their application as photosensitizer in solar cells
Source: Microb Cell Fact. 2021 Feb 10;20:41. doi: 10.1186/s12934-021-01531-4 (PMC7876818; doi:10.1186/s12934-021-01531-4)
Supplement: Supplementary file 5 — Additional file 5: Dataset S5. Phylogenetic analysis of strain UYP1. A Neighbor joining tree was constructed using 16S rRNA nucleotide sequences of fifteen Pedobacter type strains retrieved by EZBioCloud (highlighted in bold) and fifteen strains retrieved by RDP and downloaded from NCBI e-servers, as most similar in sequence with UYP1. Pedobacter sp. UYP1 phylogenetic position is indicated by an arrow. Accession numbers for all the strains are detailed in brackets. [file 12934_2021_1531_MOESM5_ESM.pptx]

## Slide 1
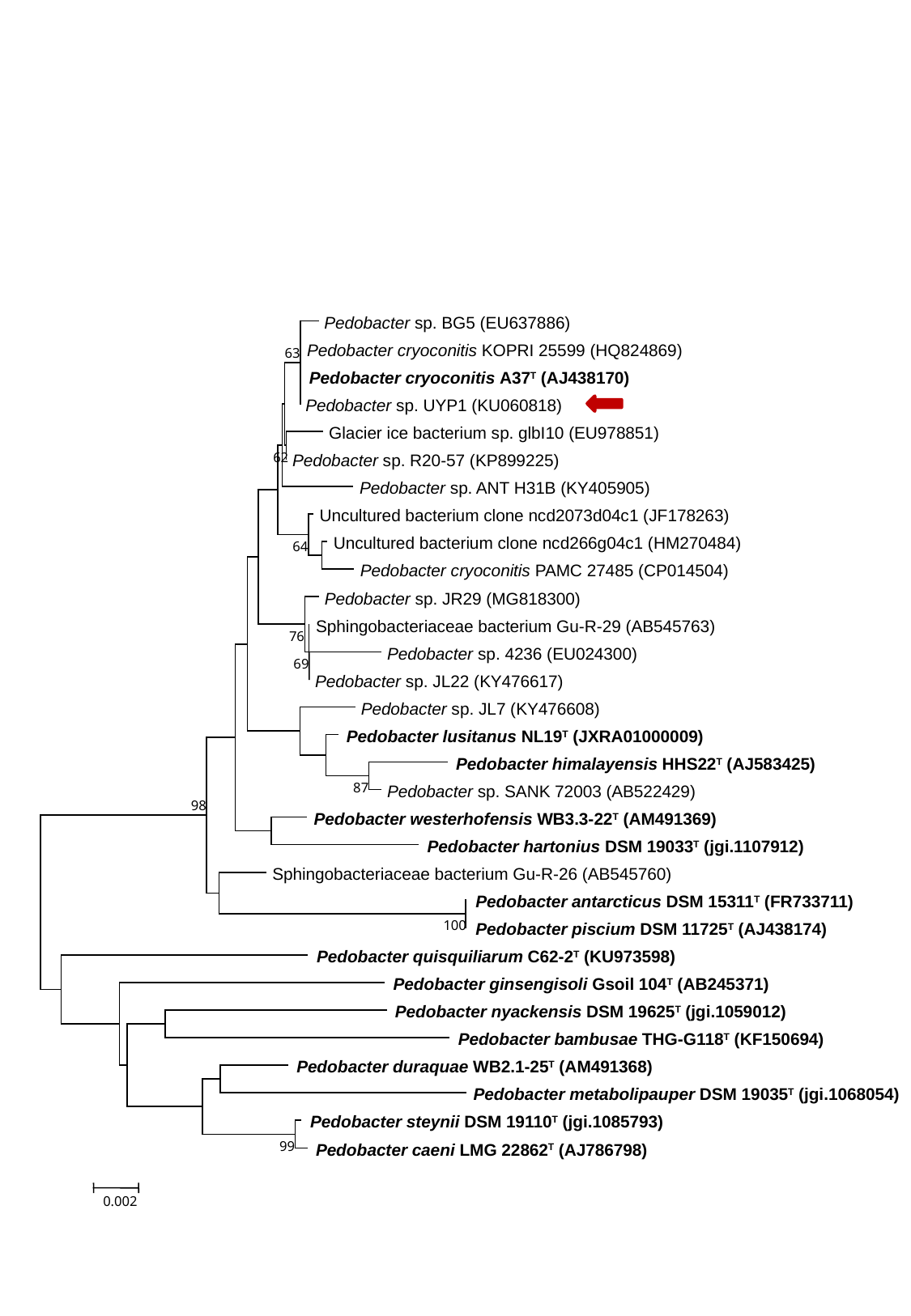

Pedobacter sp. BG5 (EU637886)
 Pedobacter cryoconitis KOPRI 25599 (HQ824869)
63
 Pedobacter cryoconitis A37T (AJ438170)
 Pedobacter sp. UYP1 (KU060818)
 Glacier ice bacterium sp. glbI10 (EU978851)
62
 Pedobacter sp. R20-57 (KP899225)
 Pedobacter sp. ANT H31B (KY405905)
 Uncultured bacterium clone ncd2073d04c1 (JF178263)
 Uncultured bacterium clone ncd266g04c1 (HM270484)
64
 Pedobacter cryoconitis PAMC 27485 (CP014504)
 Pedobacter sp. JR29 (MG818300)
 Sphingobacteriaceae bacterium Gu-R-29 (AB545763)
76
 Pedobacter sp. 4236 (EU024300)
69
 Pedobacter sp. JL22 (KY476617)
 Pedobacter sp. JL7 (KY476608)
 Pedobacter lusitanus NL19T (JXRA01000009)
 Pedobacter himalayensis HHS22T (AJ583425)
87
 Pedobacter sp. SANK 72003 (AB522429)
98
 Pedobacter westerhofensis WB3.3-22T (AM491369)
 Pedobacter hartonius DSM 19033T (jgi.1107912)
 Sphingobacteriaceae bacterium Gu-R-26 (AB545760)
 Pedobacter antarcticus DSM 15311T (FR733711)
100
 Pedobacter piscium DSM 11725T (AJ438174)
 Pedobacter quisquiliarum C62-2T (KU973598)
 Pedobacter ginsengisoli Gsoil 104T (AB245371)
 Pedobacter nyackensis DSM 19625T (jgi.1059012)
 Pedobacter bambusae THG-G118T (KF150694)
 Pedobacter duraquae WB2.1-25T (AM491368)
 Pedobacter metabolipauper DSM 19035T (jgi.1068054)
 Pedobacter steynii DSM 19110T (jgi.1085793)
99
 Pedobacter caeni LMG 22862T (AJ786798)
0.002
